# Survival of patellofemoral arthroplasty is influenced by patient characteristics, surgical factors and surgeon experience: A systematic review

**DOI:** 10.1002/jeo2.70868

**Published:** 2026-08-27

**Authors:** Jean‐Marie Philippeau, Franck Rémy, EKA, Jacobus H. Müller, Ahmed Elsaifi, Mo Saffarini, Bruno Violante, Pawel Skowronek, Reha Tandogan, Patrick Le Couteur, Guillaume Demey

**Affiliations:** ^1^ Clinique ELSAN Santé Atlantique Nantes Saint‐Herblain France; ^2^ Clinique de Saint‐Omer ELSAN Blendecques France; ^3^ ReSurg Nyon Switzerland; ^4^ Lyon‐Ortho‐Clinic Clinique de la Sauvegarde, Ramsay Santé Lyon France; ^5^ ReSurg Nyon Switzerland; ^6^ Orthopedic Department and Center for Knee and Hip Replacement and Traumatology Hospital Isola Tiberina – Gemelli Isola Rome Italy; ^7^ Orthopaedic and Trauma Department Żeromski Specialist Hospital Krakow Poland; ^8^ Ortoklinik & Çankaya Orthopedics Ankara Türkiye; ^9^ Department of Orthopedics & Traumatology Halic University Istanbul Türkiye

**Keywords:** Kaplan–Meier survival, patellofemoral arthroplasty, robot

## Abstract

**Purpose:**

While patellofemoral arthroplasty (PFA) offers advantages in preserving the tibiofemoral joint, debate remains regarding ideal patient indications and actual revision rates. The purpose of this systematic review was to identify and critically appraise comparative studies reporting survival of PFA, and to evaluate how variation in key confounding variables influences survival outcomes.

**Methods:**

Following PRISMA guidelines and PROSPERO registration, MEDLINE®, EMBASE® and Epistemonikos were searched on 23 February 2026 for comparative studies reporting PFA survival with ≥5‐year follow‐up. Data extracted included author, cohort size, implant type, follow‐up, Kaplan–Meier survival and number at risk. Effective numbers‐at‐risk and survival probabilities at each event time were reconstructed to estimate the hazard ratio (HR) for revision risk between groups. Risk of bias was assessed using Joanna Briggs Institute checklists.

**Results:**

Nine studies were included: seven retrospective clinical cohorts (1071 knees) and two registry studies (9021 knees). Registry survival was 96% at 2 years, 83%–88% at 5–6 years and 73%–76% at 10–11 years (*I* ^2^, 38.7%). Comparative analyses from clinical studies suggested that several factors, including body mass index (BMI), patellar height, robotic assistance and surgeon experience, may influence revision risk; however, most studies found no statistically significant difference, and some were in disagreement.

**Conclusion:**

Survival of PFA ranges from 73% to 76% at 10–11 years, and appears to be influenced by some non‐modifiable patient factors (age, BMI and patellar height), and modifiable surgical factors (robotic assistance and surgeon experience). These trends cannot be confirmed because of conflicting findings between eligible studies, which could be due to variability of follow‐ups (5–30 years), differences in survival endpoints (any revision or conversion to TKA), as well as the inconsistent thresholds for age and ratios of patellar height.

**Level of Evidence:**

Level IV.

**Abbreviations**
BMI: body mass index
C: cutting (inlay)
CI: confidence interval
COI: conflicts of interest
HR: hazard ratio
KM: Kaplan–Meier
n.r.: not reported
OA: osteoarthritis
PF: patellofemoral
PFA: patellofemoral arthroplasty
PFOA: patellofemoral osteoarthritis
PRISMA: Preferred Reporting Items for Systematic Reviews and Meta‐analyses
PROSPERO: International Prospective Register of Systematic Reviews
R: resurface (onlay)
TKA: total knee arthroplasty

## INTRODUCTION

Patellofemoral arthroplasty (PFA) has been shown to yield outcomes equivalent to or exceeding those of total knee arthroplasty (TKA) in appropriately selected patients with isolated patellofemoral osteoarthritis (PFOA) [1]. By replacing only the worn trochlear groove and preserving the tibiofemoral joint, PFA offers potential advantages by maintaining native joint stability, normal knee kinematics and proprioception [7]. Despite these benefits, controversy persists regarding the ideal indications [1], revision rates reported in clinical versus registry studies [3], and the superiority of trochlear cutting (inlay) versus trochlear resurfacing (onlay) implants [9].

A previous systematic review [23] of 23 cohort studies (1326 PFAs) analysed Kaplan–Meier (KM) survival using a regression line approach, reporting survival of 91.7% at 5 years, 83.3% at 10 years, 74.9% at 15 years and 66.6% at 20 years. Using the same method, survival from registry data (2495 PFAs) was 84.7% at 5 years and 71.4% at 10 years. A second systematic review [3] compared failure modes reported by 47 clinical studies (1990 PFAs) and 3 registry studies, highlighting that the main reasons for revision in clinical studies were progression of osteoarthritis (OA) and surgical error, whereas in registry studies, they were pain and aseptic loosening. Although previous systematic reviews have provided valuable insights into PFA survival and failure modes, they are more than seven years old and primarily limited to the comparison of clinical versus registry data or of specific implant brands.

The purpose of this systematic review was to identify and critically appraise studies reporting survival of PFA, and to evaluate how variation in key confounding variables influences survival outcomes. This systematic review provides an updated, comprehensive analysis of PFA survival to better inform clinical decision‐making. It evaluates the multifactorial impact of patient characteristics, surgical techniques, implant design and surgeons' experience levels.

## MATERIALS AND METHODS

The protocol for this systematic review was submitted to PROSPERO prior to commencement (CRD420250650781) and conforms to the principles outlined in the handbook of the Cochrane Collaboration, along with the guidelines established by the Preferred Reporting Items for Systematic Reviews and Meta‐analyses (PRISMA 2020) [17].

### Search strategy

An electronic literature search was conducted using Embase, Medline and Epistemonikos for articles published since inception up to 23 February 2026, using the keywords ‘patellofemoral arthroplasty’ OR ‘patello‐femoral arthroplasty’ OR ‘patellofemoral replacement’ OR ‘patello‐femoral replacement’ (Supporting Information).

### Study selection

Inclusion criteria comprised English or French studies reporting a minimum 5‐year survival for isolated PFA—with or without adjuvant ligament procedures—stratified by patient characteristics, surgical technique, implant design or surgeon experience. Studies were excluded if they were reviews, case reports, technical notes, editorials, letters or conference proceedings. Two researchers (J.H.M. and A.E.) independently screened the titles and abstracts of all records using an online tool (Rayyan) [16]. Full‐text review of studies meeting the eligibility criteria in the initial screening was performed by two researchers (J.H.M. and A.E.), and any disagreements were discussed between the researchers until consensus was achieved.

### Data extraction and quality assessment

Data extraction was performed independently by two researchers (J.H.M. and A.E.). Disagreements in the documented values were resolved by simultaneous review of the source data in question by both researchers. The following data were extracted from the included studies: author, year of publication, journal, implant type, experimental and control groups, number of patients and knees and follow‐up period. KM survival estimates were extracted from the text or from plots.

The methodological quality of the eligible studies was assessed by two researchers (J.H.M. and A.E.) according to the Joanna Briggs Institute Checklist to appraise the reporting quality [14] (Table 1). Where there was disagreement between the researchers, consensus was achieved by discussion and review.

**Table 1:** Methodological quality assessment.

Questions	Interpretation
Q1: Were there clear criteria for inclusion in the case series?	1. Authors have to describe inclusion and exclusion criteria. 2. Look for indications/contra‐indications for PFA. 3. How are patients selected for a specific treatment, e.g., choice of performing robotic‐assisted vs conventional PFA.
	Single‐arm study: A yes for Q1 or Q2. Double‐arm study: A yes for (Q1 or Q2). A yes for Q3.
Q2. Was the condition measured in a standard, reliable way for all participants included in the case series?	Did the authors assess the degree of osteoarthritis, e.g., radiographic? Did the authors quantify the degree of trochlear dysplasia? Yes, if 1 out of 2 is specified.
Q3. Were valid methods used for identification of the condition for all participants included in the case series?	Is the PFA model clearly identified, e.g., 2nd generation inlay prosthesis?
Q4. Did the case series have consecutive inclusion of participants?	1. Should describe the inclusion period or state ‘consecutive inclusion’. 2. State the number of potential participants and quantify the number excluded with reasons.
Q5. Did the case series have complete inclusion of participants?	Was there a ‘high’ proportion of patients lost to follow‐up? Guideline >40% at 10 years/>50% at 15 years/>60% at 20 years
Q6. Was there clear reporting of the demographics of the participants in the study?	Baseline (at index surgery) demographics: Age, BMI, Sex, number of bilateral cases, side, etc. Yes for at ≥3 of the parameters.
Q7. Was there clear reporting of clinical information of the participants?	Stating comorbidities at baseline: ASA score, CCI score, etc. Yes if comorbidities are stated
Q8. Were the outcomes or follow‐up results of cases clearly reported?	Survival or revision rate: 1. Proportion with confidence interval; HR 2. KM curve with confidence interval; HR 3. CIF with confidence interval
	Specify endpoint for survival or revision
Q9. Was there clear reporting of the presenting site(s)/clinic(s) demographic information?	1. Is the setting described, e.g., private hospital for elective surgeries? 2. Is the population described in terms of race, socio‐economic status?
	1/2 is yes.
Q10. Was statistical analysis appropriate?	If KM/CIF is reported: Are censored patients indicated, and are the number at risk reported at each follow‐up point? If revision rates: Number at risk, and proportion with confidence interval.

Abbreviations: ASA, American Society of Anesthesiologists; BMI, body mass index; CCI, charlson comorbidity index; CIF, cumulative incidence function; HR, hazard ratio; KM, Kaplan–Meier; PFA, patellofemoral arthroplasty.

### Data synthesis and analysis

For each included study, the assessed subgroups, number of knees per group, follow‐up period, KM survival (with its 95% CI if reported) and number at risk (if reported) were tabulated. KM survival curves that did not report tabulated survival proportions or numbers‐at‐risk were digitised to extract survival estimates and time points at each time step of the curve.

For clinical studies that reported time‐to‐event outcomes only in KM curves, hazard ratios (HRs) were estimated using the methods and Excel tool described and developed by Tierney et al. [21]. Effective numbers‐at‐risk and survival probabilities at each event time were reconstructed from the digitised KM curves for experimental and control groups. It was necessary to estimate the number of patients who were event‐free at the start of an interval, as well as the number of events, the number censored (assuming a constant rate) and the number at risk during the interval. This allowed calculation of the observed events minus the log‐rank of the expected events (O‐E), the log‐variance (V) and the HR for each interval. The reconstructed data were then used to estimate the O‐E, V and HR by combining the estimates across time intervals [21].

For registry studies, implant survival was estimated using pooled KM survival curves in a non‐parametric random‐effects model as described by Combescure et al. [6]. A distribution‐free summary implant survival curve was generated by extending the product‐limit estimator to aggregated time‐to‐event data derived from published KM curves. Survival probabilities were extracted from registry‐specific KM curves at successive time points. A random‐effects framework was applied to the conditional implant survival probabilities to account for between‐registry heterogeneity, using an extension of the DerSimonian and Laird methodology for multiple outcomes [20]. Between‐study variability was quantified using the *I* ^2^ statistic calculated from arcsine‐transformed conditional survival estimates.

Statistical analyses were performed using RStudio 2026.01.0 Build 392 (Posit Software, PBC) and R version 4.5.2 (R: A Language and Environment for Statistical Computing. R Foundation for Statistical Computing, Vienna, Austria.). A *p* value of <0.05 was deemed statistically significant.

### Deviations from the published protocol

The protocol registered with PROSPERO specified that eligible studies should be prospective or retrospective in design, either comparative or single‐series, with a minimum follow‐up of 5 years. Included studies were required to clearly and objectively report functional outcomes, patient‐reported outcomes, complications and/or survival related to isolated PFA, with or without concomitant procedures involving the PF ligaments. The predefined outcomes of interest were progression of OA to the tibiofemoral joint, functional outcomes, patient‐reported outcomes, complication rates and implant survival.

The formal literature search and screening revealed variable and inconsistent use of instruments to assess functional and patient‐reported outcomes. Consequently, the authors elected to include and analyse only studies that stratified survival outcomes according to preoperative patient characteristics, surgical techniques or geographic location.

## RESULTS

**Figure 1:**
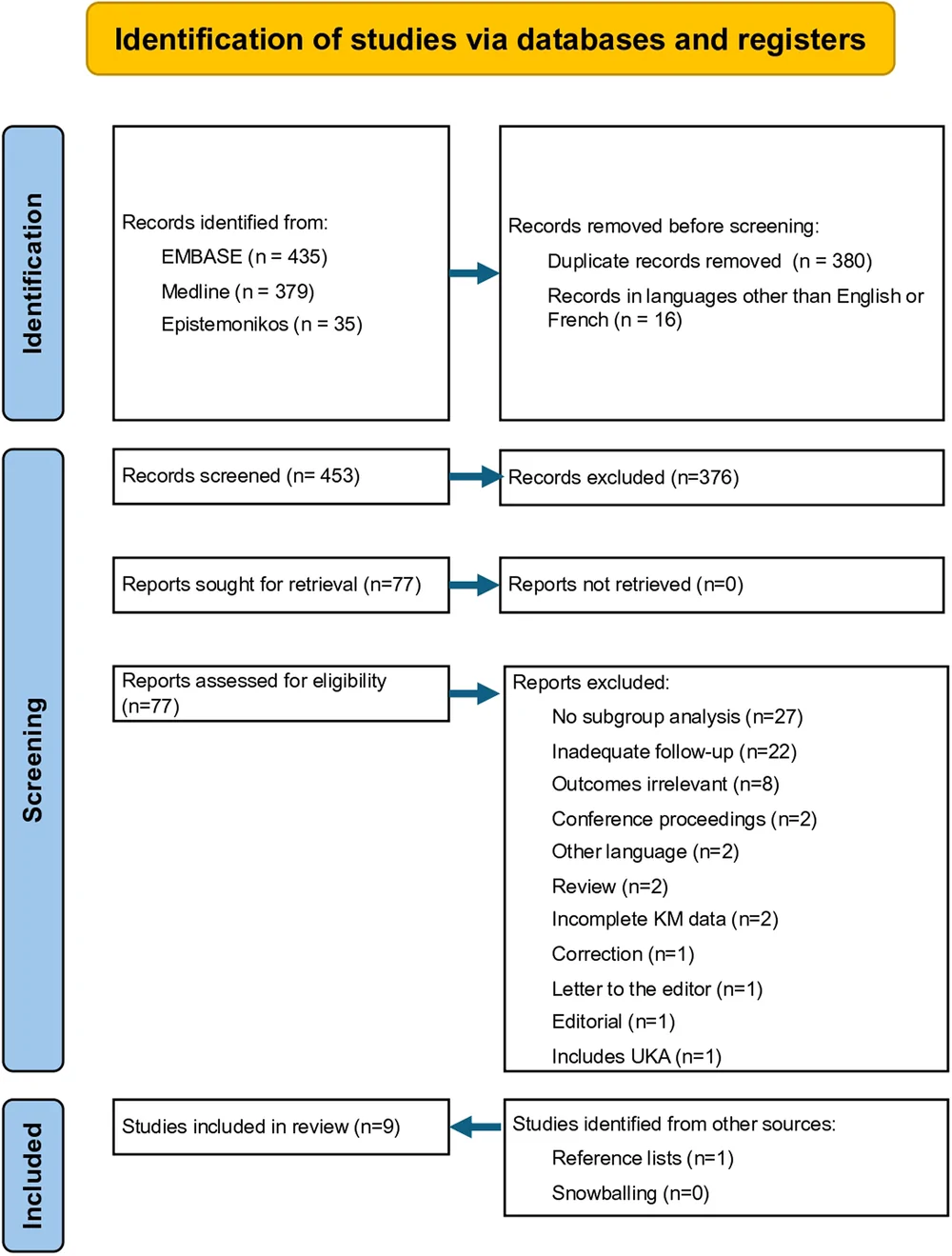
Study screening and selection PRISMA flowchart. PRISMA, Preferred Reporting Items for Systematic Reviews and Meta‐analyses; UKA, unicompartmental knee arthroplasty.

The electronic literature search returned 849 records, of which 380 were duplicates, 16 were in languages other than English or French, and 376 were excluded during title and abstract screening. Of the remaining 77 records, 69 were excluded based on their full texts (see Supporting Information). An additional record was identified in the reference lists of the remaining eight records, leaving nine studies eligible for data extraction (Figure 1) [4, 5, 8, 10, 11, 18, 19, 24, 25].

The nine eligible studies were published between 2010 and 2026, representing a total of 10,083 knees (Table 2). There were seven retrospective clinical studies (three with cohort sizes of 74–75 [5, 8, 25] and four with cohort sizes of 163–482 [4, 10, 18, 24]) and two registry‐based studies (with cohort sizes of 2150 and 6862 [11, 19]). In the nine eligible studies, the endpoint used to calculate KM survival was all‐cause revision in six studies [10, 11, 18, 19, 24, 25], conversion to TKA in two studies [4, 8], and both endpoints in only one study [5].

**Table 2:** Comparative study findings on the demographics and survival of PFA.

									Cohort characteristics				Survival		
1st Author, year	Journal	Design	Country	COI	Funding	Inclusion period	PFA type	Brand	Knees	Age, mean	Age, min	Age, max	Time point	Survival	Subgroup analyses
Saidy, 2026	*J Arthroplasty*	Registry	Australia	None	None	2015–2022	C & R	Restoris MCK trochlear implant, Stryker; Journey trochlear component, Smith & Nephew; NexGen/Gender Solutions, Zimmer Biomet; Genesis II/Journey, Smith & Nephew; Persona/Gender Solutions, Zimmer Biomet	2150	57			6 years	88.4% 87.3%	Conventional (*n* = 1481) Robotic assisted (*n* = 669)
Rasmussen, 2025	*AMA Network Open*	Population‐based Cohort study	Denmark	Yes	Yes	2008–2015	C & R	Avon 96% & 23%; Cartier 0% & 6.5%; Hemicap 0% & 4.0%; Journey 1.1% & 18%; LCS 0% & 8.5%; Sigma 0% & 9.0%; Vanguard 2.6% & 9.5%; Wave 0% & 12%; Zimmer 0.4% & 9.5%; Not recorded 0% and 3.4%	482	61 and 57			6 years	92% 74%	Trial surgeons (*n* = 274) Non‐trial surgeons (*n* = 208)
Ziedas, 2025	*J Orthop*	Retrospective	USA	None	None	2014–2022	C & R	Gender Solutions Patello‐Femoral Joint System, Zimmer Biomet; Mako system, Stryker	75	57.0 and 51.2			9 years	100% 84%	Conventional (*n* = 19) Robot assisted (*n* = 56)
Katzman, 2024	*Arch Orthop Trauma Surg*	Retrospective	USA	Yes	None	2011–2021	C & R	n.r.	184	54.4	24	86	11 years	75% 65% 60% 77%	Trochlear cutting (*n* = 89) Trochlear resurfacing (*n* = 95) Conventional (*n* = 90) Robotic assisted (*n* = 94)
Bernard, 2021	*Knee Surg Sports Traumatol*	Retrospective	USA	None	n.r.	2004–2015	C	AvonTM, Stryker & KneeTec, Tornier	163	55.8			10 years 12 years 12 years 10 years 10 years 10 years 10 years	77% 72% 90% 86% 76% 74% 78%	Overall (163) Insall Salvati ≥1.2 (*n* = 94) Insall Salvati <1.2 (*n* = 59) Caton Deschamps ≥1.3 (*n* = 18) Caton Deschamps <1.3 (*n* = 135) Blackburne Peel >1.0 (*n* = 48) Blackburne Peel ≤1.0 (*n* = 105)
Lewis, 2020	*Clin Orthop Relat Res*	Registry	Australia	None	None	2000–2016	n.r.	n.r. (registry)	6862				11 years 9 years 2 years 6 years 6 years 10 years 11 years 8 years	70.6% 81.1% 95.4% 85.1% 74.2% 83.1% 72.8% 75.6%	Australia (*n* = 3282) New Zealand (*n* = 466) Canada (*n* = 431) Kaiser Permanente (USA) (*n* = 419) Finland (*n* = 76) Norway (*n* = 433) Sweden (*n* = 517) Netherlands (*n* = 1228)
Bohu, 2019	*Knee*	Retrospective	France	Yes	Yes	1997–2015	C	HermesTM, Ceraver Osteal	74	59.6	31.3	82.1	~19 years	66% 87% 35% 87%	<60 years conversion to TKA (*n* = 42) >60 years conversion to TKA (*n* = 32) <60 years any revision (*n* = 42) >60 years any revision (*n* = 32)
de Deugd, 2017	*J Arthroplasty*	Retrospective	USA	Yes	n.r.	2002–2013	C	AvonTM, Stryker	75	51	36	81	5 years	100% 95%	Iwano Grade I (*n* = 21) Iwano Grade II–IV (*n* = 54)
van Jonbergen, 2010	*J Arthroplasty*	Retrospective	Netherlands	None	None	1976–2005	R	Richards II, Smith & Nephew	181	52			30 years	75% 82% 71% 76% 75% 75% 78% 79% 68%	Isolated primary PFOA (*n* = 138) Posttraumatic PFOA (*n* = 22) PFOA with previous realignment (*n* = 21) ≤50 years (*n* = 85) >50 years (*n* = 96) Women (*n* = 114) Men (*n* = 67) BMI ≤ 30 kg/m^2^ BMI > 30 kg/m^2^

Abbreviations: BMI, body mass index; C, cutting; COI, conflicts of interest; KM, Kaplan–Meier; n.r., not reported; PFA, patellofemoral arthroplasty; R, resurface.

^a^ Sixty‐two of the 89 inlay performed with image‐based robotic assistance; 32 of the 95 onlays were performed with imageless robotic assistance.

^b^ Study reported KM cumulative revision rates.

**Figure 2:**
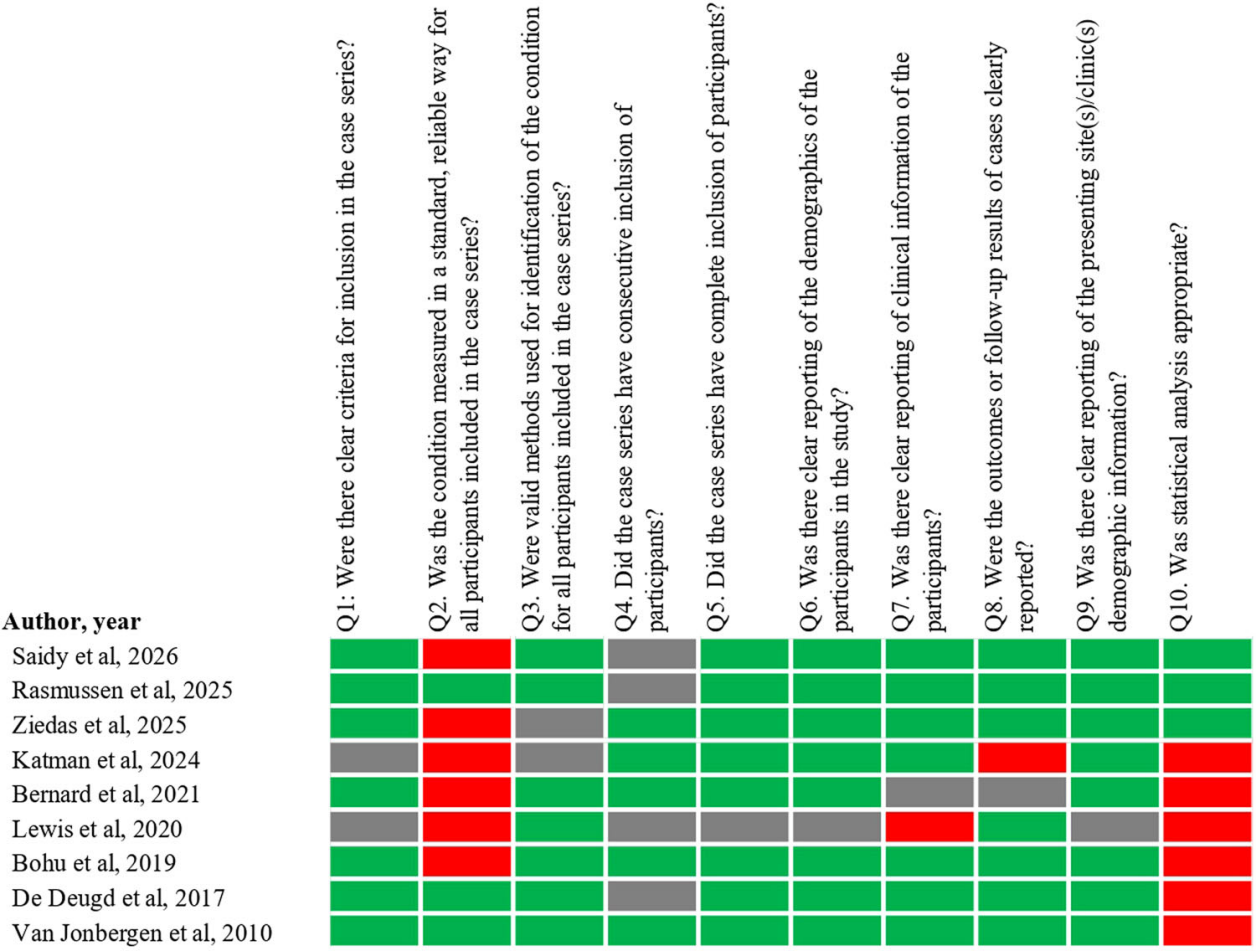
Risk of bias assessment of included studies.

The risk of bias was moderate to high across all nine eligible studies: only three adequately reported statistical analyses [18, 19, 25], and only three reported standardised measurement of the condition [8, 18, 24]. For the remaining domains, the methodological quality was adequate (Figure 2).

### Evidence from clinical studies

The seven retrospective clinical studies stratified PFA survival by patient characteristics [4, 5, 8, 24], implant design [10], robotic assistance [10, 25] and surgeon experience [18] (Table 3).

**Table 3:** Risk ratios for revision of PFA based on patient, patellofemoral and surgical characteristics.

				Group A				Group B						
	Author, year	Endpoint	Time point	Group	*n*	Survival [Number revised]	Number at risk [Time to revision]	Group	*n*	Survival [Number revised]	Number at risk [Time to revision]	Hazard ratio	95% CI	In favour
Patient characteristics														
Age	Bohu, 2019	Conversion to TKA	17 years	<60 years	40	66%	17.6	≥60 years	32	87%	18.7	2.78	0.98–7.86	n.s.
	Bohu, 2019	All‐cause revision	17 years	<60 years	40	35%	1.9	≥60 years	32	87%	3.7	3.88	1.67–9.00	≥60 years
	van Jonbergen, 2010	All‐cause revision	30 years	≤50 years	85	[20 (24%)]	[8.5 ± 8.5]	>50 years	96	[24 (25%)]	[8.8 ± 7.3]	1.20	0.60–2.10	n.s.
Sex	van Jonbergen, 2010	All‐cause revision	30 years	Women	114	[29 (25%)]	[7.1 ± 6.1]	Men	67	[15 (22%)]	[11.6 ± 9.8]	0.70	0.30–1.30	n.s.
BMI	van Jonbergen, 2010	All‐cause revision	30 years	BMI ≤ 30 kg/m^2^	124	[26 (21%)]	[9.4 ± 8.7]	BMI > 30 kg/m^2^	57	[18 (32%)]	[7.5 ± 6.1]	2.10	1.20–4.00	BMI ≤ 30 kg/m^2^
Indications	de Deugd, 2017	Conversion to TKA	5 years	Iwano I	21	100%	10.9	Iwano II–IV	54	95%	25.1	0.25	0.01–7.31	n.s.
	van Jonbergen, 2010	All‐cause revision	30 years	Group I	138	[34 (25%)]	[8.6 ± 7.8]	Group II	22	[4 (18%)]	[9.7 ± 5.7]	0.70	0.20–2.20	n.s.
	van Jonbergen, 2010	All‐cause revision	30 years	Group I	138	[34 (25%)]	[8.6 ± 7.8]	Group III	21	[6 (29%)]	[8.2 ± 10]	1.70	0.70–4.20	n.s.
Patellar height	Bernard, 2021	Conversion to TKA	12 years	Insall–Salvati >1.2	94	64%	19.2	Insall–Salvati ≤1.2	95	90%	27.5	2.76	1.30–5.93	Insall‐Salvati ≤1.2
	Bernard, 2021	Conversion to TKA	10 years	Caton–Deschamps ≥1.3	18	86%	2.7	Caton–Deschamps <1.3	135	69%	16.4	0.77	0.20–3.04	n.s.
	Bernard, 2021	Conversion to TKA	10 years	Blackburne–Peel >1.0	48	74%	6.3	Blackburne–Peel ≤1.0	105	69%	12.8	1.46	0.57–3.74	n.s.
Implant design	Katzman, 2024	All‐cause revision	11 years	Trochlear cutting	89	75%	11.2	Trochlear resurfacing	95	65%	10.4	0.75	0.41–1.49	n.s.
Robotic assistance	Saidy, 2026	All‐cause revision	6 years	Robot assisted	669	87%	44	Conventional	1481	88%	249	0.90	0.62–1.29	n.s.
	Ziedas, 2025	All‐cause revision	9 years	Robot assisted	56	84%	16.7	Conventional	19	100%	6.7	3.86	0.66–22.50	n.s.
	Katzman, 2024	All‐cause revision	11 years	Robot assisted	94	77%	32.6	Conventional	90	60%	24.3	0.51	0.27–0.96	Robot assistance
Surgeon experience	Rasmussen, 2025	All‐cause revision	6 years	Trial surgeon	274	92%	56.9	Non‐trial surgeon	208	74%	34.7	0.29	0.17–0.48	Trial surgeon

*Note*: van Jonbergen Groups: Group I, Isolated primary PFOA (*n* = 138); Group II, Posttraumatic PFOA (*n* = 22); Group III, PFOA with previous realignment (*n* = 21).

Abbreviations: BMI, body mass index; CI, confidence interval; PFOA, patellofemoral osteoarthritis; TKA, total knee arthroplasty.

The effect of age was investigated in two studies using different thresholds. Bohu et al. [5] found that patients aged under 60 had a higher risk of revision. This effect was statistically significant for all‐cause revisions (HR: 3.88, 95% CI: 1.67–9.01; *p* = 0.002) but was on the borderline for conversion to TKA (HR: 2.78, 95% CI: 0.98–7.86; *p* = n.s.). Van Jonbergen et al. [24] found that patients aged under 50 had no significant difference in risk of all‐cause revision (HR: 1.2, 95% CI: 0.6–2.1; *p* = n.s.).

The effect of sex was only investigated by van Jonbergen et al. [24], who found no significant association with risk of all‐cause revision, despite a slight trend favouring men (HR: 0.70, 95% CI: 0.30–1.30; *p* = n.s.).

The effect of BMI was also only investigated by van Jonbergen et al. [24], who found a significantly increased risk of all‐cause revision in knees with BMI > 30 kg/m^2^ (HR: 2.10, 95% CI: 1.20–4.00; *p* = 0.016).

The effect of indications for surgery on revision risk was investigated in two studies using different classifications. DeDeugd et al. [8] found that knees with PFOA of Iwano Grade I had a higher risk of conversion to TKA compared to Iwano Grades II–IV, though the difference was not statistically significant (HR: 0.25, 95% CI: 0.01–7.31; *p* = n.s.). van Jonbergen et al. [24] found no significant difference in all‐cause revision over 30 years of follow‐up between isolated primary PFOA and either post‐traumatic PFOA (HR: 0.7, 95% CI: 0.2–2.2; *p* = n.s.) or secondary PFOA following previous realignment procedures (HR: 1.7, 95% CI: 0.7–4.2; *p* = n.s.).

The effect of patellar height on the risk of conversion to TKA was investigated only by Bernard et al. [4] using three different indices. The risk was significantly greater when using a threshold for patella alta of Insall–Salvati ratio >1.2 (HR: 2.76, 95% CI: 1.30–5.93; *p* = 0.008), but not significant when using either Caton–Deschamps index ≥1.3 (HR: 0.77, 95% CI: 0.20–3.04; *p* = n.s.) or Blackburn–Peel index >1.0 (HR: 1.46, 95% CI: 0.57–3.74; *p* = n.s.).

The effect of implant design on the risk of all‐cause revision was investigated only by Katzman et al. [10]. The risk was lower for trochlear cutting (inlay) versus trochlear resurfacing (onlay) implants, but the difference was not statistically significant (HR: 0.75, 95% CI: 0.41–1.49; *p* = n.s.).

The effect of robotic assistance on the risk of all‐cause revision was investigated in two clinical studies. Katzman et al. [10] found that robotic‐assisted procedures demonstrated a significantly reduced risk (HR: 0.51, 95% CI: 0.27–0.96; *p* = 0.038). Conversely, Ziedas et al. [25] found that robotic‐assisted procedures demonstrated a greater risk, but the difference was not statistically significant (HR: 3.86, 95% CI: 0.66–22.5; *p* = n.s.).

The effect of surgeon experience on the risk of all‐cause revision was investigated only by Rasmussen et al. [18]. The risk was significantly lower for PFAs performed by surgeons who had received focused PFA training prior to participating in an RCT (trial surgeons) versus those who had not (non‐trial surgeons) (HR: 0.29, 95% CI: 0.17–0.48; *p* < 0.001).

### Evidence from registries

**Figure 3:**
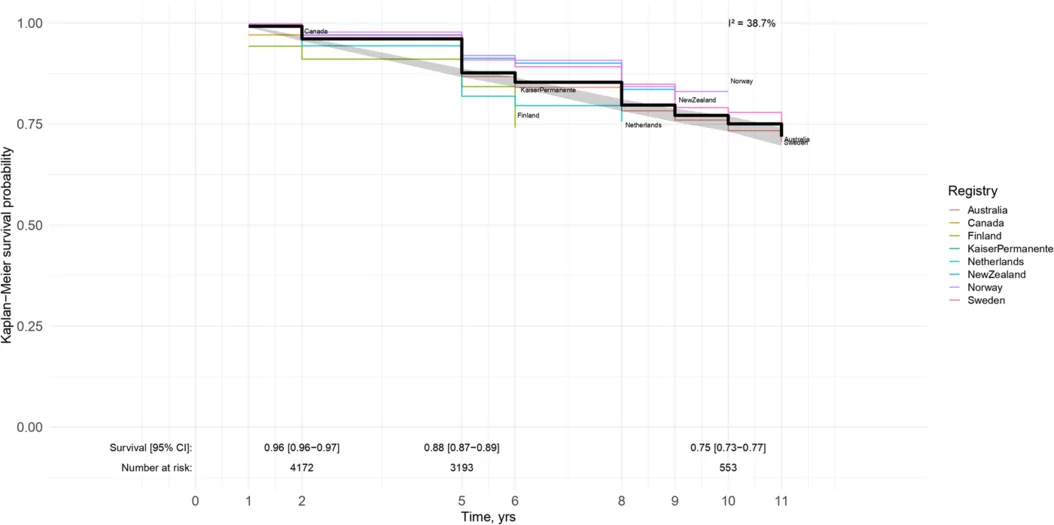
Pooled survival curves from registry data.

The two registry‐based studies stratified PFA survival by country [11] and robotic assistance [19]. Lewis et al. [11] analysed survival using all‐cause revision as endpoint across eight registries from Australia, New Zealand, Canada, Sweden, Finland, Norway, the Netherlands and the United States [11]. Anonymised data were collected for PFAs performed between January 2000 and December 2016, or where registry data collection began later, from the earliest recorded implantation date to December 2016. The number of included PFAs between registries ranged from 76 to 3282, and the follow‐up ranged from 2 to 11 years. Pooling of survival data from the different registries had moderate heterogeneity (*I* ^2^, 38.7%) and revealed overall pooled survivals of 96% at 2 years (number‐at‐risk, 4172), 88% at 5 years (number‐at‐risk, 3193), and 75% at 10 years (number‐at‐risk, 553) (Figure 3). Survival varied across different countries. Long‐term survival (9–11 years) was highest in Norway (83.1%), followed by New Zealand (81.1%), and lowest in Sweden (72.8%) and Australia (70.6%). Mid‐term survival (6–8 years) was highest in the Kaiser Permanente database (USA) (85.1%), but lowest in the Netherlands (75.6%) and Finland (74.2%).

Saidy analysed survival using all‐cause revision as endpoint from the Australian registry (AOANJRR) for 2150 PFAs performed between 2015 and 2022 [19], with a mean age of 57 years at index surgery and a follow‐up of 6 years. The cohort included 1481 conventional and 669 robot‐assisted PFAs. At 6 years, survival was 88% in the conventional group (number at risk 249) and 87% in the robot‐assisted group (number at risk 44), but our comparative analysis revealed no statistically significant difference (HR: 0.90, 95% CI: 0.62–1.29; *p* = n.s.).

## DISCUSSION

The present systematic review found nine studies that investigated factors that influence survival of PFA. One study compared survival across national registries, and found considerable differences between countries, both in the mid‐term (>11%) and long‐term (>12%). The remaining eight studies investigated the effects of non‐modifiable patient factors (age, sex, BMI, indication and patellar height) and modifiable surgical factors (implant design, robotic assistance and surgeon experience), of which five reported a significant influence of one factor, while the remaining three found no influence. It is worth noting that only one factor (robotic assistance) was investigated by three independent studies with conflicting findings, while two factors (age and indications) were each investigated by two studies, and five factors (sex, BMI, patellar height, implant design and surgeon experience) were each investigated by one study. It is therefore difficult to conclude on the influence of most factors, as they were only investigated by one or two independent studies. Moreover, these studies may not be directly comparable due to the large range of follow‐ups reported (5–30 years), different endpoints to calculate survival (any revision or conversion to TKA), as well as the inconsistent thresholds for age [5, 24], and ratios of patellar height [4].

The registry analysis by Lewis et al. [11], which combined data from eight national arthroplasty registries, demonstrated survival ranging from 70% to 95% depending on country and follow‐up. Although registry studies provide robust estimates of revision rates due to their large sample sizes and comprehensive capture of revision procedures, they frequently lack detailed clinical information such as radiographic severity of PFOA, PF morphology or surgical indications.

Of the three studies that stratified survival rates based on robotic‐assisted versus conventional PFA, only one reported significantly better implant survival at 11 years (77% vs. 60%) [10], while one found no difference at 6 years (87% vs. 88%) [19], and the other had a large inverted but non‐significant difference for an imbalanced cohort (56 vs. 19) at 9 years (84% vs. 100%) [25]. Findings from these studies should be interpreted with caution, as two had small cohorts (<100 knees per treatment arm) that resulted in wide confidence intervals and therefore imprecision [10, 25], whereas the remaining study [19] had a large initial cohort (2150 knees), but only a small proportion at risk (13%) at the final follow‐up point. A non‐comparative study by Noyes et al. [15] reported that robotic‐assisted PFA allowed precise trochlear implant alignment in younger, active patients, with 80% of knees returning to low‐impact sports, 87% achieving substantial clinical benefit, and 89% of patients reporting satisfaction over a mean follow‐up of 5.3 years. Batailler et al. [2] found that robotic‐assisted PFA, whether image‐based or image‐free, provided comparable functional outcomes to conventional techniques, while improving patellar tilt.

Of the two studies that stratified survival based on patient characteristics, only one study [5] found a significantly lower survival at 17 years using all‐cause revision as endpoint for patients <60 (35% vs. 87%), but no significant difference using conversion to TKA as endpoint (66% vs. 87%). The other study [24] reported no difference in revision rates at 30 years for patients aged over or under 50, but reported significantly higher revision rates for BMI > 30 kg/m^2^ (32% vs. 21%). Martínez‐Sañudo et al. [12] reported that BMI > 30 was associated with worse post‐operative pain, lower satisfaction and a higher likelihood of revision, despite generally good functional outcomes. In line with this, Marullo et al. [13] demonstrated that obese patients had a markedly higher failure rate (20% vs. 4.2%), primarily due to progression of tibiofemoral OA, although functional improvements were similar between groups. Conversely, Tishelman et al. [22] found no significant differences in functional outcomes or revision rates between obese and non‐obese patients.

The findings of this systematic review should be interpreted with the following considerations and limitations in mind. First, only nine eligible studies could be included and were retrospective in nature; three had cohorts of only 74 or 75 knees. This limits the level of evidence to a level of III–IV and increases the risk of selection bias. Second, the included studies had inconsistent levels of detail in patient characteristics, included a variety of PFA designs and generations, had different endpoint definitions for survival, and different follow‐up durations. Moreover, analyses on the effect of sex, BMI, implant design and surgeon experience relied on single‐study findings. Other key patellofemoral factors such as trochlear morphology, alignment or patellofemoral biomechanics were not evaluated in the included studies, which limits the clinical relevance of the conclusions of this systematic review. Therefore, findings comparing survival between comparators should be interpreted with caution, as they do not necessarily control for all confounding variables. Third, several studies included relatively small sample sizes and may therefore be underpowered to reliably detect differences between groups. Fourth, reports on KM survival analyses were incomplete, and often did not report the number‐at‐risk or the 95% CIs. This required reconstruction of KM survival curves through digitalisation, and estimation of the number‐at‐risk to calculate HRs using the methods of Tierney et al. [21]. Fifth, although no formal analysis of publication bias was performed, it cannot be ruled out given the variable and small number of studies that could be included in this systematic review. Finally, comparisons of survival between registry‐based and single‐series clinical studies are susceptible to confounding due to selection, performance and measurement biases; single‐series clinical studies may include healthier patients treated at highly resourced, specialised centres, which would artificially inflate survival outcomes compared with the unselected, real‐world populations captured in registries. Despite these limitations, the present review provides a comprehensive overview of the currently available comparative evidence on PFA.

## CONCLUSION

Survival of PFA ranges from 73% to 76% at 10–11 years, and appears to be influenced by some non‐modifiable patient factors (age, BMI and patellar height) and modifiable surgical factors (robotic assistance and surgeon experience). These trends cannot be confirmed because of conflicting findings between eligible studies, which could be due to variability of follow‐ups (5–30 years), differences in survival endpoints (any revision or conversion to TKA), as well as the inconsistent thresholds for age and ratios of patellar height.

## CONTRIBUTORS OF EKA

Jacobus H. Müller, Ahmed Elsaifi, Mo Saffarini, Bruno Violante, Pawel Skowronek and Reha Tandogan.

## AUTHOR CONTRIBUTIONS

**Jean‐Marie Philippeau**: Funding acquisition; conceptualisation; investigation; validation; writing—review and editing. **Franck Rémy**: Conceptualisation; writing—review and editing. **EKA**: Investigation; statistical analysis; software; writing—original draft. **Patrick Le Couteur**: Conceptualisation; writing—review and editing. **Guillaume Demey**: Conceptualisation; investigation; validation; writing—review and editing.

## FUNDING INFORMATION

The authors have no funding to report.

## CONFLICT OF INTEREST STATEMENT

J.H.M. member of editorial boards for *Knee Surgery, Sports Traumatology, Arthroscopy* (KSSTA) and *BMC Musculoskeletal Disorders*. The other authors declare no conflicts of interest.

## ETHICS STATEMENT

The authors have nothing to report.

## Supporting information

Supporting File 1.

## Data Availability

Upon reasonable request, the corresponding author can provide access to the data used for all analyses.
