# Supplementary material for: Survival of patellofemoral arthroplasty is influenced by patient characteristics, surgical factors and surgeon experience: A systematic review
Source: J Exp Orthop. 2026 Aug 27;13(3):e70868. doi: 10.1002/jeo2.70868 (PMC13521518; doi:10.1002/jeo2.70868)
Supplement: Supplementary file 1 [file JEO2-13-e70868-s001.docx]

**Search strategy**

*MEDLINE & Embase (Royal Society of Medicine Engine)*

"patellofemoral arthroplasty"[All Fields]

OR

"patello-femoral arthroplasty"[All Fields]

OR

"patellofemoral replacement"[All Fields]

OR

"patello-femoral replacement"[All Fields]

*Epsitemonikos*

"patellofemoral arthroplasty"[Title/Abstract]

OR

"patello-femoral arthroplasty"[ Title/Abstract]

OR

"patellofemoral replacement"[ Title/Abstract]

OR

"patello-femoral replacement"[ Title/Abstract]

**Supplementary material – Full text screening.**

The electronic literature search returned 771 records, of which 341 were duplicates, 16 were in languages other than English or French, and 355 were excluded during title and abstract screening. Of the remaining 59 records, 22 had inadequate follow-up [1,3,5,7,8,12,13,19,21,27,28,31,36,37,41-43,45,48,49,53,54], 22 were not comparative [2,4,6,9,10,14-18,22,23,25,26,29,32-35,38,50,52], 4 reported irrelevant outcomes [20,24,30,51], 2 were conference proceedings [39,47], 1 was a correction [40], 1 was a letter to the editor [11], 1 was a editorial [44] and 1 included UKA without stratification [46].

**References**

1.	Ackroyd CE, Chir B (2005) Development and early results of a new patellofemoral arthroplasty. Clin Orthop Relat Res 436 (436):7-13. doi:doi:10.1097/01.blo.0000171914.94503.d1

2.	Ackroyd CE, Newman JH, Evans R, Eldridge JD, Joslin CC (2007) The Avon patellofemoral arthroplasty: five-year survivorship and functional results. J Bone Joint Surg Br 89 (3):310-315. doi:doi:10.1302/0301-620X.89B3.18062

3.	Ajnin S, Buchanan D, Arbuthnot J, Fernandes R (2018) Patellofemoral joint replacement - Mean five year follow-up. Knee 25 (6):1272-1277. doi:doi:10.1016/j.knee.2018.08.014

4.	Argenson JN, Flecher X, Parratte S, Aubaniac JM (2005) Patellofemoral arthroplasty: an update. Clin Orthop Relat Res 440:50-53. doi:doi:10.1097/01.blo.0000187061.27573.70

5.	Argenson JN, Guillaume JM, Aubaniac JM (1995) Is there a place for patellofemoral arthroplasty? Clin Orthop Relat Res (321):162-167. doi:doi:

6.	Baker JF, Caborn DN, Schlierf TJ, Fain TB, Smith LS, Malkani AL (2020) Isolated Patellofemoral Joint Arthroplasty: Can Preoperative Bone Scans Predict Survivorship? J Arthroplasty 35 (1):57-60. doi:doi:10.1016/j.arth.2019.08.021

7.	Batailler C, Putzeys P, Lacaze F, Vincelot-Chainard C, Fontalis A, Servien E, Lustig S (2023) Patellofemoral Arthroplasty Is an Efficient Strategy for Isolated Patellofemoral Osteoarthritis with or without Robotic-Assisted System. J Pers Med 13 (4). doi:doi:10.3390/jpm13040625

8.	Butler JE, Shannon R (2009) Patellofemoral arthroplasty with a custom-fit femoral prosthesis. Orthopedics 32 (2):81. doi:doi:<http://dx.doi.org/10.3928/01477447-20090201-09>

9.	Cartier P, Sanouiller JL, Khefacha A (2005) Long-term results with the first patellofemoral prosthesis. Clin Orthop Relat Res 436 (436):47-54. doi:doi:10.1097/01.blo.0000171918.24998.d1

10.	Clement ND, Howard TA, Immelman RJ, MacDonald D, Patton JT, Lawson GM, Burnett R (2019) Patellofemoral arthroplasty versus total knee arthroplasty for patients with patellofemoral osteoarthritis: equal function and satisfaction but higher revision rate for partial arthroplasty at a minimum eight years' follow-up. Bone Joint J 101 (1):41-46. doi:doi:10.1302/0301-620X.101B1.BJJ-2018-0654.R2

11.	Coleridge S (2002) The Lubinus patellofemoral arthroplasty: a five- to ten-year prospective study. J Bone Joint Surg Br 84 (2):306; author reply 306-307. doi:doi:

12.	Cozzarelli NF, Khan IA, Arshi A, Sherman MB, Lonner JH, Fillingham YA (2024) Return to Sport After Unicompartmental Knee Arthroplasty and Patello-Femoral Arthroplasty. J Arthroplasty 39 (8):1988-1995 e1985. doi:doi:10.1016/j.arth.2024.02.004

13.	Dahm DL, Kalisvaart MM, Stuart MJ, Slettedahl SW (2014) Patellofemoral arthroplasty: outcomes and factors associated with early progression of tibiofemoral arthritis. Knee Surg Sports Traumatol Arthrosc 22 (10):2554-2559. doi:doi:10.1007/s00167-014-3202-3

14.	de Winter WE, Feith R, van Loon CJ (2001) The Richards type II patellofemoral arthroplasty: 26 cases followed for 1-20 years. Acta Orthop Scand 72 (5):487-490. doi:doi:10.1080/000164701753532826

15.	Gadeyne S, Besse JL, Galand-Desme S, Lerat JL, Moyen B (2008) [Results of self-centering patellofemoral prosthesis: a retrospective study of 57 implants]. Rev Chir Orthop Reparatrice Appar Mot 94 (3):228-240. doi:doi:10.1016/j.rco.2007.06.001

16.	Hernigou P, Caton J (2014) Design, operative technique and ten-year results of the Hermes patellofemoral arthroplasty. Int Orthop 38 (2):437-442. doi:doi:10.1007/s00264-013-2158-0

17.	Hoogervorst P, de Jong RJ, Hannink G, van Kampen A (2015) A 21% conversion rate to total knee arthroplasty of a first-generation patellofemoral prosthesis at a mean follow-up of 9.7 years. Int Orthop 39 (9):1857-1864. doi:doi:10.1007/s00264-015-2941-1

18.	Imhoff AB, Feucht MJ, Bartsch E, Cotic M, Pogorzelski J (2019) High patient satisfaction with significant improvement in knee function and pain relief after mid-term follow-up in patients with isolated patellofemoral inlay arthroplasty. Knee Surg Sports Traumatol Arthrosc 27 (7):2251-2258. doi:doi:10.1007/s00167-018-5173-2

19.	Jagadeesh N, Sales-Fernandez R, Pammi S, Kariya A (2022) Functional Outcomes, Survival Rate, and Complications of Patellofemoral Arthroplasty: Mid-Term Results From Independent Center. Cureus 14 (11):e31945. doi:doi:10.7759/cureus.31945

20.	Joseph MN, Achten J, Parsons NR, Costa ML, Collaborators PATT (2020) The PAT randomized clinical trial. Bone Joint J 102 (3):310-318. doi:doi:10.1302/0301-620X.102B3.BJJ-2019-0723.R1

21.	Kazarian GS, Tarity TD, Hansen EN, Cai J, Lonner JH (2016) Significant Functional Improvement at 2 Years After Isolated Patellofemoral Arthroplasty With an Onlay Trochlear Implant, But Low Mental Health Scores Predispose to Dissatisfaction. J Arthroplasty 31 (2):389-394. doi:doi:10.1016/j.arth.2015.08.033

22.	Konan S, Haddad FS (2016) Midterm Outcome of Avon Patellofemoral Arthroplasty for Posttraumatic Unicompartmental Osteoarthritis. J Arthroplasty 31 (12):2657-2659. doi:doi:10.1016/j.arth.2016.06.005

23.	Kooijman HJ, Driessen AP, van Horn JR (2003) Long-term results of patellofemoral arthroplasty. A report of 56 arthroplasties with 17 years of follow-up. J Bone Joint Surg Br 85 (6):836-840. doi:doi:

24.	Lewis PL, Graves SE, Cuthbert A, Parker D, Myers P (2019) What Is the Risk of Repeat Revision When Patellofemoral Replacement Is Revised to TKA? An Analysis of 482 Cases From a Large National Arthroplasty Registry. Clin Orthop Relat Res 477 (6):1402-1410. doi:doi:10.1097/CORR.0000000000000541

25.	Martinez-Lozano J, Martorell-de Fortuny L, Martin-Dominguez LA, Torres-Claramunt R, Sanchez-Soler J, Perelli S, Hinarejos P, Monllau JC (2025) Patellofemoral arthroplasty provides similar long-term survival rate and complications with better clinical outcomes compared to facetectomy for the treatment of isolated patellofemoral osteoarthritis. J Exp Orthop 12 (1):e70136. doi:doi:10.1002/jeo2.70136

26.	Martinez-Sanudo B, Fornell S, Vallejo M, Domecq G (2023) Midterm outcomes of patellofemoral arthroplasty. Rev Esp Cir Ortop Traumatol 67 (4):317-323. doi:doi:10.1016/j.recot.2022.12.016

27.	Marullo M, Bargagliotti M, Vigano M, Lacagnina C, Romagnoli S (2022) Patellofemoral arthroplasty: obesity linked to high risk of revision and progression of medial tibiofemoral osteoarthritis. Knee Surg Sports Traumatol Arthrosc 30 (12):4115-4122. doi:doi:10.1007/s00167-022-06947-y

28.	Merchant AC (2005) A modular prosthesis for patellofemoral arthroplasty: design and initial results. Clin Orthop Relat Res 436 (436):40-46. doi:doi:10.1097/01.blo.0000171917.47869.6c

29.	Middleton SWF, Toms AD, Schranz PJ, Mandalia VI (2018) Mid-term survivorship and clinical outcomes of the Avon patellofemoral joint replacement. Knee 25 (2):323-328. doi:doi:10.1016/j.knee.2018.01.007

30.	Mofidi A, Veravalli K, Jinnah RH, Poehling GG (2014) Association and impact of patellofemoral dysplasia on patellofemoral arthropathy and arthroplasty. Knee 21 (2):509-513. doi:doi:10.1016/j.knee.2013.09.009

31.	Mohammed R, Jimulia T, Durve K, Bansal M, Green M, Learmonth D (2008) Medium-term results of patellofemoral joint arthroplasty. Acta Orthop Belg 74 (4):472-477. doi:doi:

32.	Mont MA, Johnson AJ, Naziri Q, Kolisek FR, Leadbetter WB (2012) Patellofemoral arthroplasty: 7-year mean follow-up. J Arthroplasty 27 (3):358-361. doi:doi:10.1016/j.arth.2011.07.010

33.	Noyes FR, Fleckenstein CM, Nolan J (2024) Return to Sports in Patients Aged 50 Years or Younger After Robotic-Assisted Patellofemoral Arthroplasty: A 10-Year Experience Reporting High Clinical Benefits and High Patient Satisfaction With Return to an Active Lifestyle. Am J Sports Med 52 (6):1514-1526. doi:doi:10.1177/03635465241237460

34.	Odgaard A, Kappel A, Madsen F, Kristensen PW, Stephensen S, Attarzadeh AP (2022) Patellofemoral Arthroplasty Results in Better Time-weighted Patient-reported Outcomes After 6 Years than TKA: A Randomized Controlled Trial. Clin Orthop Relat Res 480 (9):1707-1718. doi:doi:10.1097/CORR.0000000000002178

35.	Pagano A, Agostinone P, Alesi D, Caputo D, Neri MP, Grassi A, Zaffagnini S (2024) Almost 79% survival rate at 10-year follow-up for the patellofemoral joint arthroplasty: An Italian prosthetic registry study. Knee Surg Sports Traumatol Arthrosc 32 (6):1525-1530. doi:doi:10.1002/ksa.12150

36.	Perrone FL, Baron S, Suero EM, Lausmann C, Kendoff D, Zahar A, Gehrke T, Citak M (2018) Patient-reported outcome measures (PROMs) in patients undergoing patellofemoral arthroplasty and total knee replacement: A comparative study. Technol Health Care 26 (3):507-514. doi:doi:10.3233/THC-181185

37.	Pogorzelski J, Rupp MC, Ketzer C, Cotic M, Lutz P, Beeck S, Imhoff AB, Feucht MJ (2021) Reliable improvements in participation in low-impact sports following implantation of a patellofemoral inlay arthroplasty at mid-term follow-up. Knee Surg Sports Traumatol Arthrosc 29 (10):3392-3399. doi:doi:10.1007/s00167-020-06245-5

38.	Rammohan R, Gupta S, Lee PYF, Chandratreya A (2019) The midterm results of a cohort study of patellofemoral arthroplasty from a non-designer centre using an asymmetric trochlear prosthesis. Knee 26 (6):1348-1353. doi:doi:10.1016/j.knee.2019.10.026

39.	Ramos P, Arteaga G, Vargas M, Naranjo J (2017) Our Experience with Patello femoral joint replacement. Orthopaedic Journal of Sports Medicine 5 (1). doi:doi:10.1177/2325967117s00027

40.	Research E-i-CoCO, Related (2023) Expression of Concern: Patellofemoral Arthroplasty Results in Better Time-weighted Patient-reported Outcomes After 6 Years Than TKA: A Randomized Controlled Trial. Clin Orthop Relat Res 481 (2):416-417. doi:doi:10.1097/CORR.0000000000002523

41.	Romagnoli S, Marullo M (2018) Mid-Term Clinical, Functional, and Radiographic Outcomes of 105 Gender-Specific Patellofemoral Arthroplasties, With or Without the Association of Medial Unicompartmental Knee Arthroplasty. J Arthroplasty 33 (3):688-695. doi:doi:10.1016/j.arth.2017.10.019

42.	Shubin Stein BE, Brady JM, Grawe B, Tuakli-Wosornu Y, Nguyen JT, Wolfe E, Voigt M, Mahony G, Strickland S (2017) Return to Activities After Patellofemoral Arthroplasty. Am J Orthop (Belle Mead NJ) 46 (6):E353-E357. doi:doi:

43.	Sisto DJ, Sarin VK (2006) Custom patellofemoral arthroplasty of the knee. J Bone Joint Surg Am 88 (7):1475-1480. doi:doi:10.2106/JBJS.E.00382

44.	Slutsky DJ (2014) The use of tables. J Wrist Surg 3 (4):219. doi:doi:10.1055/s-0034-1395165

45.	Smith AM, Peckett WR, Butler-Manuel PA, Venu KM, d'Arcy JC (2002) Treatment of patello-femoral arthritis using the Lubinus patello-femoral arthroplasty: a retrospective review. Knee 9 (1):27-30. doi:doi:10.1016/s0968-0160(01)00127-2

46.	Tauro B, Ackroyd CE, Newman JH, Shah NA (2001) The Lubinus patellofemoral arthroplasty. A five- to ten-year prospective study. J Bone Joint Surg Br 83 (5):696-701. doi:doi:10.1302/0301-620x.83b5.11577

47.	Tee WJ, Ho JPY, Abbas AA, Merican AM (2018) Treatment of isolated patellofemoral osteoarthritis with patellofemoral arthroplasty: A case series. Malaysian Orthopaedic Journal 12. doi:doi:

48.	Tishelman JC, Pyne A, Kahlenberg CA, Gruskay JA, Strickland SM (2022) Obesity Does Not Affect Patient-Reported Outcomes following Patellofemoral Arthroplasty. J Knee Surg 35 (3):312-316. doi:doi:10.1055/s-0040-1713862

49.	Utukuri MM, Khanduja V, Somayaji HS, Dowd GS (2008) Patient-based outcomes in patellofemoral arthroplasty. J Knee Surg 21 (4):269-274. doi:doi:10.1055/s-0030-1247830

50.	van Wagenberg JM, Speigner B, Gosens T, de Waal Malefijt J (2009) Midterm clinical results of the Autocentric II patellofemoral prosthesis. Int Orthop 33 (6):1603-1608. doi:doi:10.1007/s00264-009-0719-z

51.	Vella-Baldacchino M, Davies AR, Bottle A, Cobb J, Liddle AD (2025) Association Between Surgeon Volume and Patient Outcomes After Elective Patellofemoral Arthroplasty: A Population-Based Cohort Study Using Data from the National Joint Registry and Hospital Episode Statistics for England. J Bone Joint Surg Am. doi:doi:10.2106/JBJS.24.00703

52.	Wang Y, Bian Y, Qian W (2023) Long-Term clinical results of patellofemoral arthroplasty for isolated patellofemoral osteoarthritis. J Orthop Surg (Hong Kong) 31 (1):10225536231162832. doi:doi:10.1177/10225536231162832

53.	Willekens P, Victor J, Verbruggen D, Vande Kerckhove M, Van Der Straeten C (2015) Outcome of patellofemoral arthroplasty, determinants for success. Acta Orthop Belg 81 (4):759-767. doi:doi:

54.	Yassa R, Khalfaoui MY, Davies AP (2016) Are "Patellofemoral Symptoms" Truly Related to the Patellofemoral Joint? Knee Surg Relat Res 28 (1):68-74. doi:doi:10.5792/ksrr.2016.28.1.68
